# Comparing machine learning algorithms for predicting ICU admission and mortality in COVID-19

**DOI:** 10.1038/s41746-021-00456-x

**Published:** 2021-05-21

**Authors:** Sonu Subudhi, Ashish Verma, Ankit B. Patel, C. Corey Hardin, Melin J. Khandekar, Hang Lee, Dustin McEvoy, Triantafyllos Stylianopoulos, Lance L. Munn, Sayon Dutta, Rakesh K. Jain

**Affiliations:** 1 Department of Medicine/Gastroenterology Division, Massachusetts General Hospital and Harvard Medical School, Boston, MA USA; 2 Department of Medicine/Renal Division, Brigham and Women’s Hospital and Harvard Medical School, Boston, MA USA; 3 Department of Pulmonary and Critical Care Medicine, Massachusetts General Hospital and Harvard Medical School, Boston, MA USA; 4 Department of Radiation Oncology, Massachusetts General Hospital and Harvard Medical School, Boston, MA USA; 5 Biostatistics Center, Massachusetts General Hospital and Harvard Medical School, Boston, MA USA; 6 Mass General Brigham Digital Health eCare, Somerville, MA USA; 7 Cancer Biophysics Laboratory, Department of Mechanical and Manufacturing Engineering, University of Cyprus, Nicosia, Cyprus; 8 Edwin L. Steele Laboratories, Department of Radiation Oncology, Massachusetts General Hospital and Harvard Medical School, Boston, MA USA; 9 Department of Emergency Medicine, Massachusetts General Hospital and Harvard Medical School, Boston, MA USA

**Keywords:** Epidemiology, Prognosis, Predictive medicine, Machine learning

## Abstract

As predicting the trajectory of COVID-19 is challenging, machine learning models could assist physicians in identifying high-risk individuals. This study compares the performance of 18 machine learning algorithms for predicting ICU admission and mortality among COVID-19 patients. Using COVID-19 patient data from the Mass General Brigham (MGB) Healthcare database, we developed and internally validated models using patients presenting to the Emergency Department (ED) between March-April 2020 (*n* = 3597) and further validated them using temporally distinct individuals who presented to the ED between May-August 2020 (*n* = 1711). We show that ensemble-based models perform better than other model types at predicting both 5-day ICU admission and 28-day mortality from COVID-19. CRP, LDH, and O_2_ saturation were important for ICU admission models whereas eGFR <60 ml/min/1.73 m^2^, and neutrophil and lymphocyte percentages were the most important variables for predicting mortality. Implementing such models could help in clinical decision-making for future infectious disease outbreaks including COVID-19.

## Introduction

The COVID-19 pandemic has led to significant morbidity and mortality throughout the world^1^. The rapid spread of SARS-CoV-2 has provided limited time to identify factors involved in SARS-CoV-2 transmission, predictors of COVID-19 severity, and effective treatments. At the height of the pandemic, areas with high numbers of SARS-CoV-2 infections were resource-limited and forced to triage life-saving therapies such as ventilators and dialysis machines^2,3^. In this setting, identifying patients requiring intensive care or at high risk of mortality upon presentation to the hospital may help providers expedite patients to the most appropriate care setting. Additionally, novel therapies such as remdesivir have been shown to decrease the length of hospitalization when administered early in disease course, while dexamethasone has been shown to decrease COVID-19 mortality when administered later in more severe disease^4–6^. Prediction of which patients are at high risk of progression and poor outcomes can guide clinicians in treatment choices during this critical time in a patient’s disease course.

Model predictions are gaining increased interest in clinical medicine. Machine learning applications have been used to help predict acute kidney injury^7^ and septic shock^8^, amongst other outcomes in hospitalized patients. These tools have also been applied to outpatients to predict outcomes such as heart failure progression^9^. Machine learning tools can also be applied to predict outcomes such as Intensive Care Unit (ICU) admission and mortality^10^. Thus far there have been few studies that examined specific machine learning algorithms in predicting outcomes such as ICU admission/mortality in COVID-19 patients^11–15^. Given the potential utility of machine learning-based decision rules and the urgency of the pandemic, a concerted effort is being made to identify which machine learning applications are optimal for given sets of data and diseases^16^.

To address this knowledge gap, we conducted a multi-hospital cohort study to extensively evaluate the performance of 18 different machine learning algorithms in predicting ICU admission and mortality. Our goal was to identify the best prognostication algorithm using demographic data, comorbidities, and laboratory findings of COVID-19 patients who visited emergency departments (EDs) at Massachusetts General Brigham (MGB) hospitals between March and April 2020. We validated our models on a temporally distinct patient cohort that tested positive for COVID-19 and had an ED encounter between May and August 2020. We also identified critical variables utilized by the model to predict ICU admission and mortality.

## Results

### Patient characteristics

We obtained data from 10,826 patients in the multi-hospital Massachusetts General Brigham (MGB) Healthcare database, which consists of patients from academic and community hospitals, who had COVID-19 infection during the period of March and April 2020. A total of 3713 out of the 10,826 patients who tested positive for SARS-CoV-2 visited an ED. We evaluated patients based on demographics, home medications, past medical history, clinical features, and laboratory values as described in Supplementary Table 1. We excluded patients who had one or more missing dependent (outcome) variables and we imputed missing data for independent (predictor) variables. After excluding patients with missing outcome information (i.e., ICU admission and mortality information), 3597 patients remained. For temporal validation, we extracted data from the MGB healthcare database for patients who tested positive for SARS-CoV-2 between May and August 2020. During this period, 1754 out of 8013 SARS-CoV-2 positive individuals visited the ED. Similarly, after excluding patients with missing dependent/outcome variable (Supplementary Table 1), a total of 1711 patients remained.

After imputing missing independent variable data, the baseline characteristics of 3597 patients in the training dataset are listed in Table 1. The overall study population included 48.7% women, and the median age was 55 years. The number of patients admitted to the ICU within 5 days and who died within 28 days of the ED visit were 486 (13.5%) and 344 (9.6%), respectively. The temporal validation dataset included patients with similar distribution in age ≥50 years (X^2^_(4, N=3073)_ = 7.7, *p* = 0.1), gender (X^2^_(1, N=5308)_ = 0.63, *p* = 0.43) and race (X^2^_(1, N=5308)_ = 2.48, *p* = 0.11), but BMI was significantly different (X^2^_(2, N=5308)_ = 13.8, *p* = 0.001) (Supplementary Table 6). Of the 1711 patients who visited the ED, 146 (8.5%) were admitted to the ICU and 78 (4.5%) died of COVID-19.

### Comparing performance of prediction models–cross validation

**Fig. 1:**
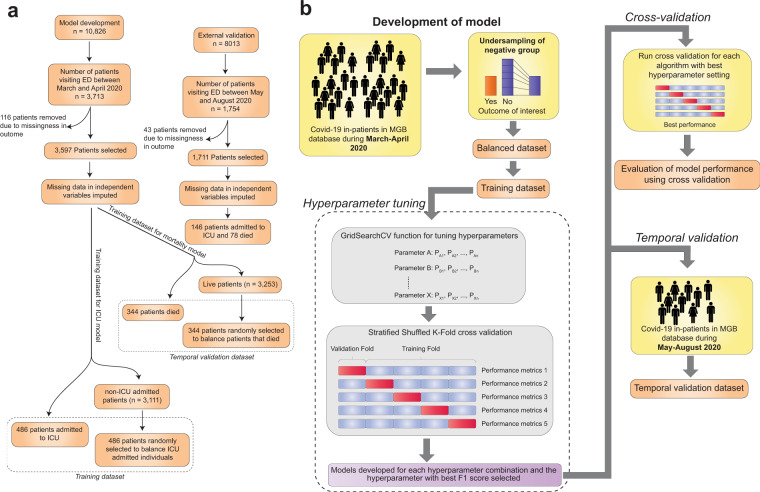
Schematic diagram representing the process of machine learning model development. **a** Flow diagram depicting steps in obtaining the training and temporal validation datasets (with patient numbers in each step). **b** The process of patient selection, dataset balancing, hyperparameter tuning, cross-validation and temporal validation are shown.

We evaluated 18 machine learning algorithms belonging to 9 broad categories, namely ensemble, Gaussian process, linear, naïve bayes, nearest neighbor, support vector machine, tree-based, discriminant analysis and neural network models Fig. 1.

**Fig. 2:**
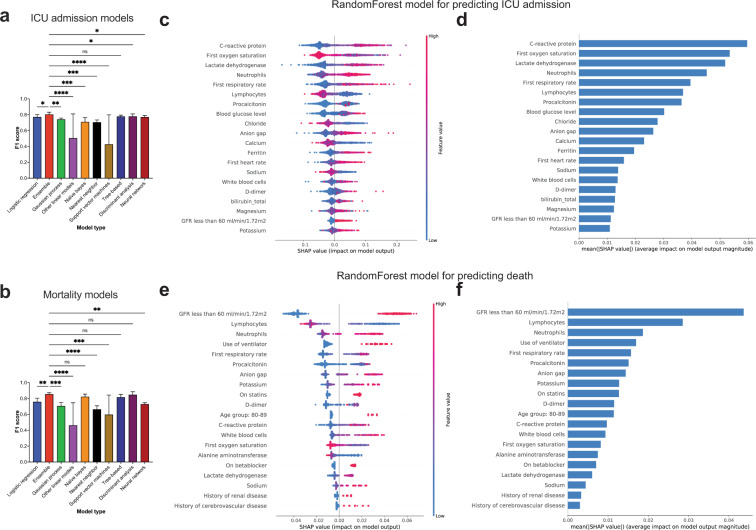
F1 score comparison and variables of importance for ICU admission and mortality prediction models. **a, b** Bar plots representing the F1 scores of ICU admission and mortality prediction models. Error bars indicate standard deviation from mean. Statistical analysis was performed using Two-stage step-up method of Benjamini, Krieger and Yekutieli test which controls for False discovery rate (FDR) during multiple comparison. *p-*value style is geometric progression - <0.03 (*), <0.002 (**), <0.0002 (***), <0.0001 (****). **c** SHAP value summary dot plot and **d** variable importance of *RandomForest* algorithm-based ICU admission model. **e** SHAP value summary dot plot and **f** variable importance of *RandomForest* algorithm-based mortality model. The calculation of SHAP values is done by comparing the prediction of the model with and without the feature in every possible way of adding the feature to the model. The bar plot depicts the mean SHAP values whereas the summary dot plot shows the impact on the model. The color of the dot represents the value of the feature and the X-axis depicts the direction and magnitude of the impact. Red colored dots represent high value of the feature and the blue represents lower value. A positive SHAP value means the feature value increases likelihood of ICU admission/mortality. For features with positive SHAP value for red dots, suggests directly proportional variable to outcome of interest and those with positive SHAP value for blue dots, suggest inverse correlation.

Comparing the ICU admission prediction models using cross validation, we observed that all ensemble-based models had mean F1 scores ≥0.8 (Table 2; Supplementary Fig. 1A–C). Specifically, the F1 score for *AdaBoostClassifier* was 0.80 (95% CI, 0.75–0.85), for *BaggingClassifier* was 0.81 (95% CI, 0.77–0.85), for *GradientBoostingClassifier* was 0.81 (95% CI, 0.77–0.85), for *RandomForestClassifier* was 0.81 (95% CI, 0.78–0.84), for *XGBClassifier* was 0.8 (95% CI, 0.76–0.84), and for *ExtraTreesClassifier* was [0.8 (95% CI, 0.76–0.84)]. In addition, *LogisticRegression* [0.77 (95% CI, 0.73–0.81)], *DecisionTreeClassifier* [0.78 (95% CI, 0.76–0.80)], *LinearDiscriminantAnalysis* [0.77 (95% CI, 0.72–0.82)], *QuadraticDiscriminantAnalysis* [0.79 (95% CI, 0.78–0.80)] and *MLPClassifier* [0.77 (95% CI, 0.74–0.8)] also had high F1 scores. In contrast, *PassiveAggressiveClassifier*, *Perceptron* and *LinearSVC* models had relatively low F1 scores. Upon performing multiple comparison analysis between all models (based on PR AUC and F1 scores), the ensemble-based models, *LinearDiscriminantAnalysis*, *MLPClassifier* and *LogisticRegression* models had similar performance characteristics (Supplementary Fig. 1A–C). By grouping the models based on their broad categories, we found that ensemble and tree-based models had significantly higher F1 scores than all other model types (Fig. 2a; details of statistical analysis in Supplementary Table 7).

We next compared the mortality prediction models using cross validation and found that all ensemble-based models had mean F1 scores higher than 0.83 (Table 3; Supplementary Fig. 1D–F). The F1 score for *AdaBoostClassifier* was 0.84 (95% CI, 0.81–0.87), *BaggingClassifier* was 0.83 (95% CI, 0.80–0.86), *GradientBoostingClassifier* was 0.86 (95% CI, 0.84–0.88), *RandomForestClassifier* was 0.87 (95% CI, 0.85–0.89), *XGBClassifier* was 0.85 (95% CI, 0.84–0.86), and *ExtraTreesClassifier* was 0.87 (95% CI, 0.85–0.89)]. In addition, *LinearDiscriminantAnalysis* [0.88 (95% CI, 0.86–0.90)], *QuadraticDiscriminantAnalysis* [0.81 (95% CI, 0.77–0.85)], *GaussianNB* [0.82 (95% CI, 0.78–0.86)] and *DecisionTreeClassifier* [0.82 (95% CI, 0.77–0.87)] also had high F1 scores. However, for mortality prediction, *LogisticRegression* [0.76 (95% CI, 0.70–0.82)] had a low F1 score compared to ensemble methods. The lowest F1 scores were for *PassiveAggressiveClassifier*, *Perceptron*, *LinearSVC* and *KNeighborsClassifier* (Table 3). Upon performing a multiple comparison analysis between all models (based on PR AUC and F1 scores), the ensemble-based models, *GaussianNB* and *LinearDiscriminantAnalysis* models had similar patterns of performance (Supplementary Fig. 1D–F). When we grouped the models based on their broad categories and compared their F1 scores, we found that ensemble-based models performed better than all other model types except naïve bayes, tree-based and discriminant analysis-based methods (Fig. 2b; details of statistical analysis in Supplementary Table 7).

For calculating expected classification error, we used the Brier score which provides the mean squared error between probability estimates and actual outcome. A lower score indicates more accurate predictions. We observed that ensemble methods had lower Brier score for ICU admission (mean Brier score <0.15 except *AdaBoostClassifier*; Table 2) and mortality prediction models (mean Brier score <0.12 except *AdaBoostClassifier*; Table 3).

### Comparing performance of prediction models–temporal validation

We then tested the temporally distinct dataset on ICU admission models and found that ensemble-based methods, had higher F1 (≥0.4) and PR AUC (≥0.5) scores compared to other methods (Table 2). Although *LogisticRegression* and *LinearDiscriminantAnalysis* models had comparable F1 scores, their PR AUC scores were lower than ensemble-based methods. When the performance of mortality models was evaluated using a temporally distinct dataset, ensemble-based methods, *LogisticRegression*, *GaussianNB*, *DecisionTreeClassifier*, and *LinearDiscriminantAnalysis* had relatively higher F1 scores (≥0.26) compared to other mortality models (Table 3). There was a more severe drop in F1 scores for temporally distinct patients for mortality prediction compared to ICU admission. For comparing classification error, we observed that the Brier score was lower for ensemble methods for both ICU admission and mortality prediction models (Tables 2 and 3). Based on calibration plots, we found that all models over-estimated the risk of disease, but ensemble-methods were closer to the true risk (Random Forest and logistic regression model plots shown - Supplementary Fig. 2).

Overall, even though performance of all machine learning models dropped in the temporal validation dataset, the ensemble models remained the best at predicting both ICU admission and mortality for COVID-19 patients.

### Critical variables for predicting ICU admission and mortality

To investigate how individual variables in the machine learning models impact outcome prediction, we performed a SHAP analysis of the random forest model as it was one of the best-performing models (based on F1 scores) among the ensemble-based models for predicting ICU admission and mortality. For the ICU admission prediction models, C-reactive protein, neutrophil percentages, lactate dehydrogenase, and first respiratory rate were directly proportional to risk of ICU admission (Fig. 2c, d), while lower oxygen saturation and lymphocyte percentages were associated with increased probability of ICU admission. For mortality prediction models, use of ventilator, estimated glomerular filtration rate (eGFR) less than 60 ml/min/1.72 m^2^, high neutrophil percentage, high serum potassium, low lymphocyte percentages, and high procalcitonin were associated with higher mortality (Fig. 2e, f). To address the reduction in F1 scores for ICU admission and mortality models, we performed a SHAP analysis on the temporally distinct patients to compare the important variables for model predictions. For predicting ICU admission, the top variables remained similar to the important variables in the primary random forest model (Fig. 2c and d compared to Supplementary Fig. 3A and B). However, for predicting mortality in the temporal validation cohort, D-dimer and initial oxygen saturation became more important, while ventilator use was less important compared to the primary random forest model (Fig. 2e and f compared to Supplementary Fig. 3C and D).

## Discussion

In this study, we evaluated the ability of various machine learning algorithms to predict clinical outcomes such as ICU admission or mortality using data available from the initial ED encounter of COVID-19 patients. Based on our analysis of 18 algorithms, we found that ensemble-based methods have moderately better performance than other machine learning algorithms. Optimizing the hyperparameters (Supplementary Tables 4 and 5) enabled us to achieve the best-performing ensemble models. We also identified variables that had the largest impact on the performance of the models. We demonstrated that for predicting ICU admission, C-reactive protein, LDH, procalcitonin, lymphocyte percentage, neutrophil percentage, oxygen saturation and respiratory rate were among the top predictors, but for mortality prediction, eGFR < 60 ml/min/1.73 m^2^, use of ventilator, lymphocyte percentage, neutrophil percentage, respiratory rate, procalcitonin, serum anion gap and serum potassium were the leading predictors.

Our model detected that CRP, LDH, procalcitonin, eGFR < 60 ml/min/m^2^, serum potassium levels, advanced age and ventilator use were indicative of a worse outcome, which aligns with previous studies of ICU admission and mortality (Supplementary Table 2). Retrospective studies have shown increased procalcitonin values associated with high risk for severe COVID-19 infection^17^. The explanation for this association is not clear. Increased procalcitonin levels in COVID -19 patients can suggest bacterial coinfection but may also be a marker of hyperinflammation and/or a marker of ARDS severity^18–20^. We also found reduced kidney function as the major risk factor for mortality, however, based on the design of the current study, it is not clear whether pre-existing renal dysfunction is a causal factor for poor outcomes in COVID-19 or a consequence of more severe COVID-19 infection. This result has been revealed by two previous studies in the literature, indicating that patients with chronic kidney disease with or without dialysis have a high risk of mortality from COVID-19^21,22^. Our study also highlighted serum potassium level as an important predictor for mortality. This finding has been reported in the literature by two previous studies to our knowledge^23,24^ and one study has reported the high prevalence of hypokalemia among patients with COVID-19^25^. Potassium derangement is independently associated with increased mortality in ICU patients^26,27^. Deviations in serum potassium levels in COVID-19 patients may represent dysregulation of the renin-angiotensin system^28^ which has been suggested to also play a role in SARS-CoV pathogenesis^29^. However, there was no comparison to a hyperkalemia group in this study as in previous studies. By treating serum potassium as a continuous variable, we have identified higher serum potassium levels on presentation to be a predictor of ICU admission and mortality which maybe more reflective of impaired potassium excretion due to decreased kidney function as the cause. This finding shows that the model aligns with previously reported clinically relevant markers and also predicts new markers that emerged from our patient population.

For predicting ICU admission, the top variables remained similar in both the training and temporal validation cohorts. However, in predicting mortality in the temporal validation cohort, D-dimer and initial oxygen saturation became more important, while the ventilator use became less important than the training cohort. Though the exact reason for this change with the temporal validation cohort is uncertain, there are a number of reasons that can be speculated. Mortality for COVID-19 has decreased overtime^30^ which may have led to differences in predictive variables. The reasons for this decrease in mortality are not well understood. It occurred despite little change in patient acuity or presentation and has been attributed variously to increased adherence to standard evidenced based therapies for acute respiratory failure. Disease outcome has been reported elsewhere to have improved over time during the pandemic, possibly due to greater familiarity and comfort with COVID-19 and the development of specific treatments (chiefly remdesivir and dexamethasone) may have led to declining mortality over time^31^.

Our study utilized a multi-hospital cohort that has been developed and validated in temporarily distinct subsets. Multiple studies in the past have used machine learning methodology for the identification of clinical phenotypes in COVID-19 patients^11–13,32,33^. However, these studies were oriented toward identifying clinical features rather than determining the best machine learning algorithm for predicting clinical outcomes in this novel disease, so only a limited number of models were tested. To our knowledge, this is the first study to quantitatively and systematically compare multiple machine learning models. We demonstrated that ensemble-methods perform better than other methods in predicting ICU admission and mortality from COVID-19. Ensemble methods are meta-algorithms that combine several different machine learning techniques into one unified predictive model (Supplementary Table 3)^34^, which could explain their superior performance. We also performed hyperparameter tuning to determine the best model performance values (F1 score). By performing SHAP analysis, we showed how variables impact outcomes in black-box machine learning models. Thus, our study is consistent with previous clinical study results, revealing similar clinical predictors for ICU admission and mortality, utilizing higher-performing machine learning models.

There are a few weaknesses in our analysis. Since not all machine learning algorithms are capable of predicting probabilities (some models return decision function attribute), we were unable to calibrate the models uniformly. Therefore, we resorted to using the Brier score as a metric for determining expected classification errors, which includes discrimination and calibration. A lower Brier score indicates more accurate predictions but does not necessarily mean better calibration. During hyperparameter tuning, we considered F1-score as the primary metric for selecting best hyperparameter for each model. The drawback of using F1-score is that it does not have a good intuitive explanation. In this study, we used beta = 1 as a metric, and therefore it has been referred to as F1-score. However, based on expectations out of a model, one has to modify the F-beta score which would attach beta times as much importance to recall as to precision.

Another limitation was related to using the *k*-nearest neighbor algorithm for imputing missing values in dependent variables. This algorithm assumes that the missing value is similar to that of other patients who are more similar, based on other available features. Although this method of imputation is superior to other imputation methods, it does have a risk of data distortion^35^. Additional shortcomings of the study are associated with using a SHAP (TreeSHAP) analysis for determining variable of importance. The SHAP analysis needs to be specifically adapted for a machine learning algorithm - particularly for ensemble methods–to make it more versatile and computationally efficient.

There are a few additional limitations in our study from a clinical aspect. Some of the laboratory results may take hours to be reported, and the data may not be available until after the patient has transitioned out of the ED. This limits the utility of using these laboratory predictors in triaging patient disposition. Another limitation is that features related to the disease course prior to presentation to ED were unavailable, which limited our ability to verify rapidity of worsening symptoms. Future studies aimed at training models based on time course data might allow earlier identification of high-risk individuals.

Overall, the performance of our models on the temporal validation dataset dropped, which might be attributed to changes in management practices, evolution of SARS-CoV-2 pathogenesis, or due to the imbalanced nature of the dataset. We also observed that the F1 scores on the temporal validation cohort (imbalanced dataset) were relatively higher for ICU admission models in comparison to mortality models. This could be due changes in the important variables for predicting mortality (Fig. 2c–f, Supplementary Fig. 3). Future studies might provide a more definitive answer to the question–“How did changes instated in the ICU during the later period of pandemic affect mortality?” Changes in treatment regimens may affect the relative importance of variables over time, thereby affecting the mortality prediction of our models. Since the most important variables for predicting ICU admission did not change between the temporally different cohorts, the drop in F1 score during temporal validation might be due to the imbalanced nature of the dataset. Our cohort is based on a population from Southern New England region of United States and included two tertiary academic centers, which could also limit the versatility of the models, as resources available at these hospitals may not be available elsewhere. Larger, more expansive studies based on this framework in other cohorts would help validate our findings before clinical deployment of these models.

Our model development process and findings could be used by clinicians in gauging the clinical course, particularly ICU admission, of an individual with COVID-19 during an ED encounter. We recommend using ensemble-based methods for developing clinical prediction models in COVID-19. Our ensemble methods identified key features in patients, such as kidney function, lymphocyte percentage, neutrophil percentage, CRP and LDH, that allowed us to predict clinical outcomes. Deploying such models could augment the clinical decision-making process by allowing physicians to identify potentially high-risk individuals and adjust their treatment and triaging accordingly.

## Methods

### Study population

Patients from the Mass General Brigham (MGB) healthcare system that were positive for SARS-CoV-2 between March and August of 2020 and had an ED encounter were included. Patients either had COVID-19 prior to the index ED visit or were diagnosed during that encounter. MGB is an integrated health care system which encompasses 14 hospitals across New England in the United States. COVID-19 positive patients were defined by the COVID-19 infection status, a discretely recorded field in the Epic EHR (Epic Systems Inc., Verona, WI). The COVID-19 infection status was added automatically if a SARS-CoV-2 PCR test was positive, or by Infection Control personnel if the patient had a confirmed positive test from an outside facility. This study was approved by the MGB Institutional Review Board (IRB protocol # 2020P000964).

### Data collection and covariate selection

We queried the data warehouse of our EHR for patient-level data including demographics, comorbidities, home medications, most recent outpatient recorded blood pressure, and death date. For each hospital encounter we extracted vital signs, laboratory values, admitting service, hospital length of stay, date of first ICU admission, amongst others. We considered only the first clinical and laboratory values that were recorded after ED admission. The patient’s problem list was extracted and transformed into a comorbidity matrix by using the *comorbidity* R package^36^.

### Outcome definition

The two primary outcomes used for developing the models were ICU admission within 5 days of ED encounter and mortality within 28 days of ED encounter. The beginning of the prediction window began upon arrival to the ED.

### Model development

As described in Supplementary Table 1, we selected a reduced set of potential predictor variables from previously published literature (Supplementary Table 2). We used the same covariates in developing the ICU admission and mortality models except for ventilator use which was added to mortality models but excluded from ICU admission models. Age (10 year intervals), race (African American or other), BMI, modified Charlson Comorbidity Index^37^, angiotensin converting enzyme inhibitor/angiotensin receptor blocker (ACEi/ARB) use, hypertension (>140/90 mmHg), and eGFR <60 ml/min were treated as categorical values. Patients with missing values for the dependent variables (outcome: ICU admission or mortality information) or obviously incorrect entries (e.g., one patient was listed with respiratory rate of 75 breaths per minute) were excluded. Missing values were imputed using the *k*-nearest neighbor algorithm^38,39^. Models were developed using the patients admitted during the period of March and April 2020. For model validation, we used a temporally distinct cohort consisting of patients admitted from May through August 2020. The data set was imbalanced with significantly fewer patients who were admitted to the ICU or who died due to COVID-19 compared with those who did not. For the purpose of developing the machine learning models, we performed random undersampling of the majority class and used these balanced datasets for developing machine learning models. To rule out bias during undersampling, we compared the excluded patients of the majority class with patients who were included to ensure that none of the variables were significantly different (*p* ≥ 0.05; Supplementary Table 8). We avoided oversampling techniques to balance the datasets to prevent overfitting and to reduce computation time^40^.

A total of eighteen machine learning algorithms were tested, the descriptions of which are available in Supplementary Table 3. For every machine learning model, we used a three-step approach. First, we made models using various combinations of tunable hyperparameters which were used to control the learning process of algorithms. The hyperparameters that were adjusted depended on the algorithm (outlined in Supplementary Table 4). After developing these models for each combination of hyperparameter, we tested the performance of each of these combinations (performance metrics generated for each combination of hyperparameter––data not shown) using a cross validation technique (number of folds = 5) during which the F1 score was considered to select the best hyperparameter (Supplementary Table 5). The F1 score is a measure that unites the trade-offs of precision and recall and provides a single number that represents the utility of a classifier in predicting the minority class. For grading the performance of models, we used F1 scores as this is more applicable for datasets that are imbalanced^41^. In our case, the temporal validation dataset remained an imbalanced dataset.

### Evaluation of model performance

Model performance evaluation was done in two parts. A *StratifiedKFold* technique of cross validation was first used during model development. In this method, 20% of the patients were excluded while training the model and the excluded patients were then used to test the model. This was performed using an iterative process. Each model was evaluated by calculating the Receiver Operating Characteristic Area Under the Curve (ROC AUC), PR AUC, F1, recall, precision, balanced accuracy, and Brier scores. To calculate the 95% confidence interval, we used t_0.975, df=4_ = 2.776 based on *t*-distribution for *n* = 5. Secondly, for the temporal validation, the cohort of patients who presented to the ED between May and August 2020 was used (Supplementary Table 6).

### Model interpretation using Shapley values

For explaining the models, SHAP feature importance was reported based on Shapley values^42^, details of which are outlined in the Supplementary Methods. SHAP values are useful to explain “black-box” machine learning models which are otherwise difficult to interpret. SHAP values for each patient feature explain the intensity and direction of impact on predicting the outcome.

### Reporting summary

Further information on research design is available in the Nature Research Reporting Summary linked to this article.

## Supplementary information

Supplementary Information

Reporting Summary

## Data Availability

The clinical data used in this study belongs to MGB healthcare and restrictions apply to the availability of these data. Qualified researchers affiliated with the Mass General Brigham (MGB) may apply for access to these data through the MGB institutional review board.

## Code availability

The programming code for R and Python are available upon request addressed to the corresponding authors: Sayon Dutta, MD (sdutta1@partners.org) and Rakesh K. Jain, PhD (jain@steele.mgh.harvard.edu). Data cleaning and processing were performed with R (R Core Team, version 3.6.3) using the *tidyverse* and *comorbidity* packages (for cross referencing ICD-10 problem list diagnoses to their corresponding Charlson comorbidities)^36,43,44^. Machine learning model development was done using Python (details in Supplementary Methods)^45–48^.
