# Supplementary material for: Comparing machine learning algorithms for predicting ICU admission and mortality in COVID-19
Source: NPJ Digit Med. 2021 May 21;4:87. doi: 10.1038/s41746-021-00456-x (PMC8140139; doi:10.1038/s41746-021-00456-x)
Supplement: Supplementary file 1 — Supplementary Information [file 41746_2021_456_MOESM1_ESM.pdf]

Supplementary Materials

Supplementary methods:

Model interpretation using Shapley values

For explaining the models, SHAP feature importance was reported based on Shapley values. *TreeExplainer* based SHAP values were used to demonstrate the impact of covariates on model prediction. SHAP analysis was performed for best performing model for ICU admission and mortality prediction. We also performed SHAP analysis on models developed using temporal validation dataset to explain important variables for ICU admission and mortality prediction later on during the pandemic.

Softwares and packages

For processing the clinical data, dplyr and tableone packages in R were used[35,36,43]. After processing, model development was performed in Python (version 3.8; Python Software Foundation) using open-source packages including *sklearn*, *xgboost*, *imblearn*, *matplotlib* and *shap* packages[45-48]. *GridsearchCV* and *cross_val_score* functions were used to identify the best hyperparameters[40]. For comparing model types, GraphPad prism software (version 8.4.3) was used.

Supplementary figure 1. Matrix plots showing differential model performance

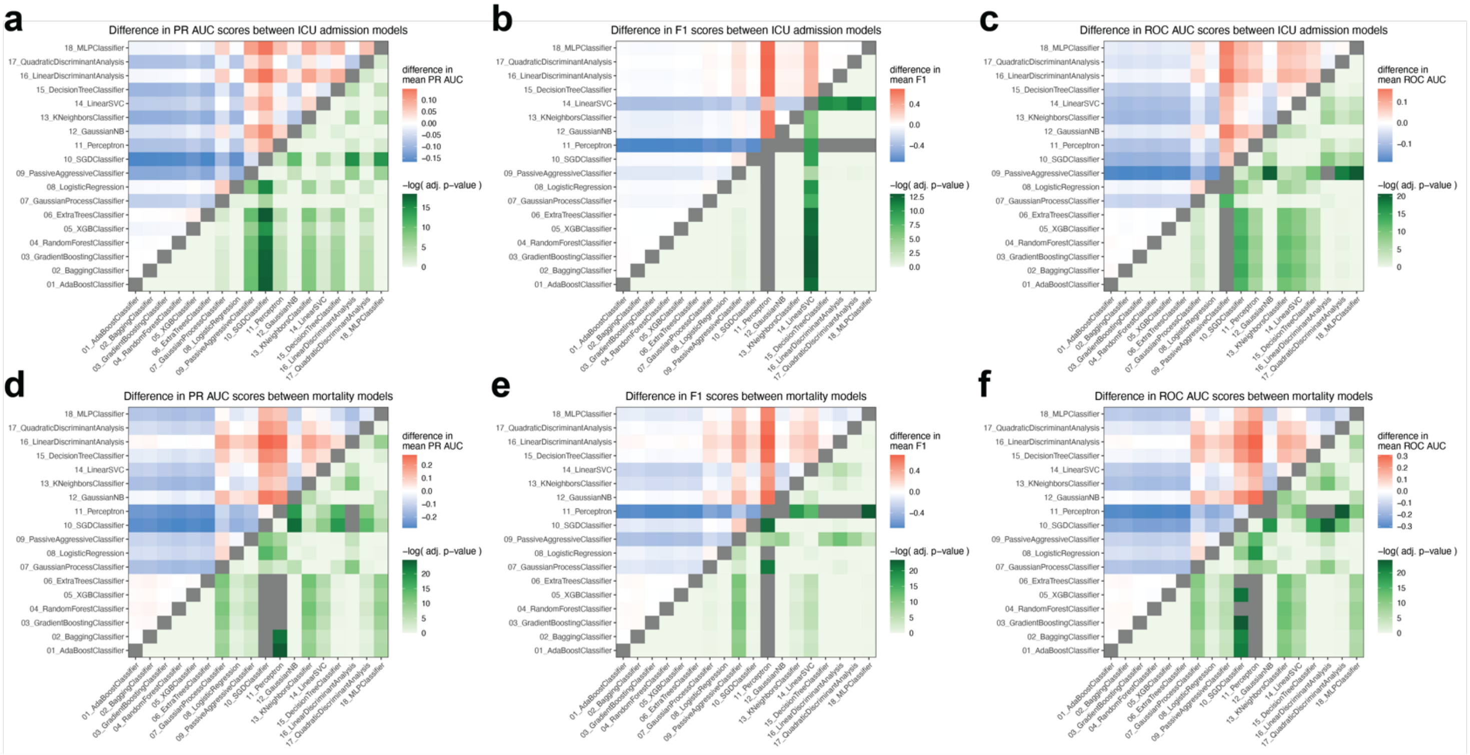

Supplementary figure 1. Matrix plots showing differential model performance of ICU admission prediction models on the basis of (A) PR AUC, (B) F1, (C) ROC AUC scores, and of mortality prediction models on the basis of (D) PR AUC, (E) F1, (F) ROC AUC scores. The 3 performance metrics of all 18 machine learning models are compared using mean score differences and adjusted p values (based on multiple values generated during cross validation). The upper left half shows the difference in mean and the botton right half represents the negative logarithm of adjusted P values. Darker red or blue color represents more difference between the mean scores and a darker green color represents its significance.

Supplementary figure 2. ROC AUC, PR AUC and calibration plots

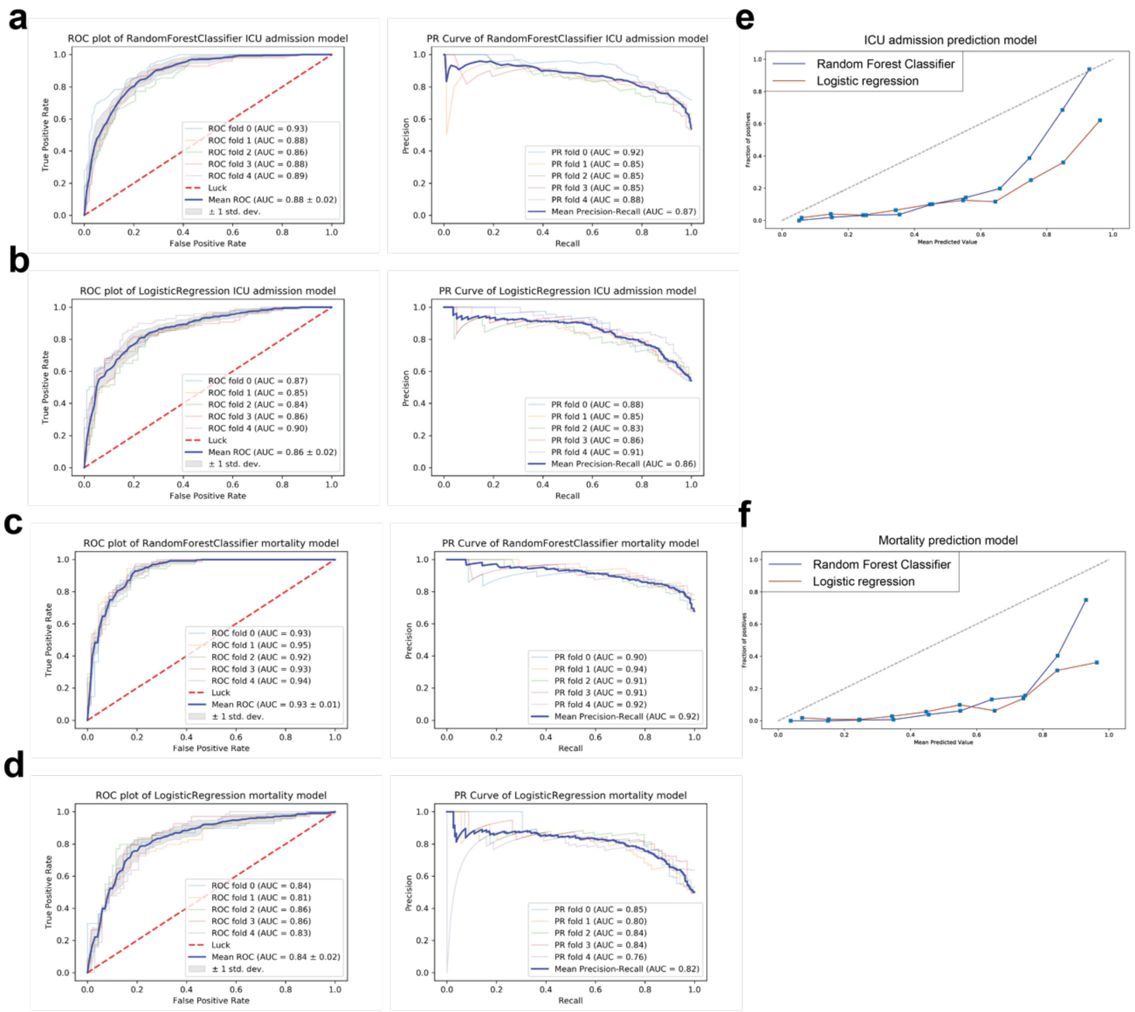

Supplementary figure 2. ROC AUC, PR AUC and Calibration plots. ROC AUC and PR AUC plots shown while performing cross validation of RandomForestClassifier and LogisticRegression based models for ICU admission (A-B) and mortality prediction (C-D). Curves plotted for for each resampling of cross validation (n = 5) on training dataset and average curve plotted in dark blue. Calibration plots of ICU admission model, using RandomForestClassifier and LogisticRegression (E), and mortality prediction models, using RandomForestClassifier and LogisticRegression (F), plotted based on probability estimates of temporal validation patients.

Supplementary figure 3. Variable of importance for ICU admission and mortality in temporally distinct patients

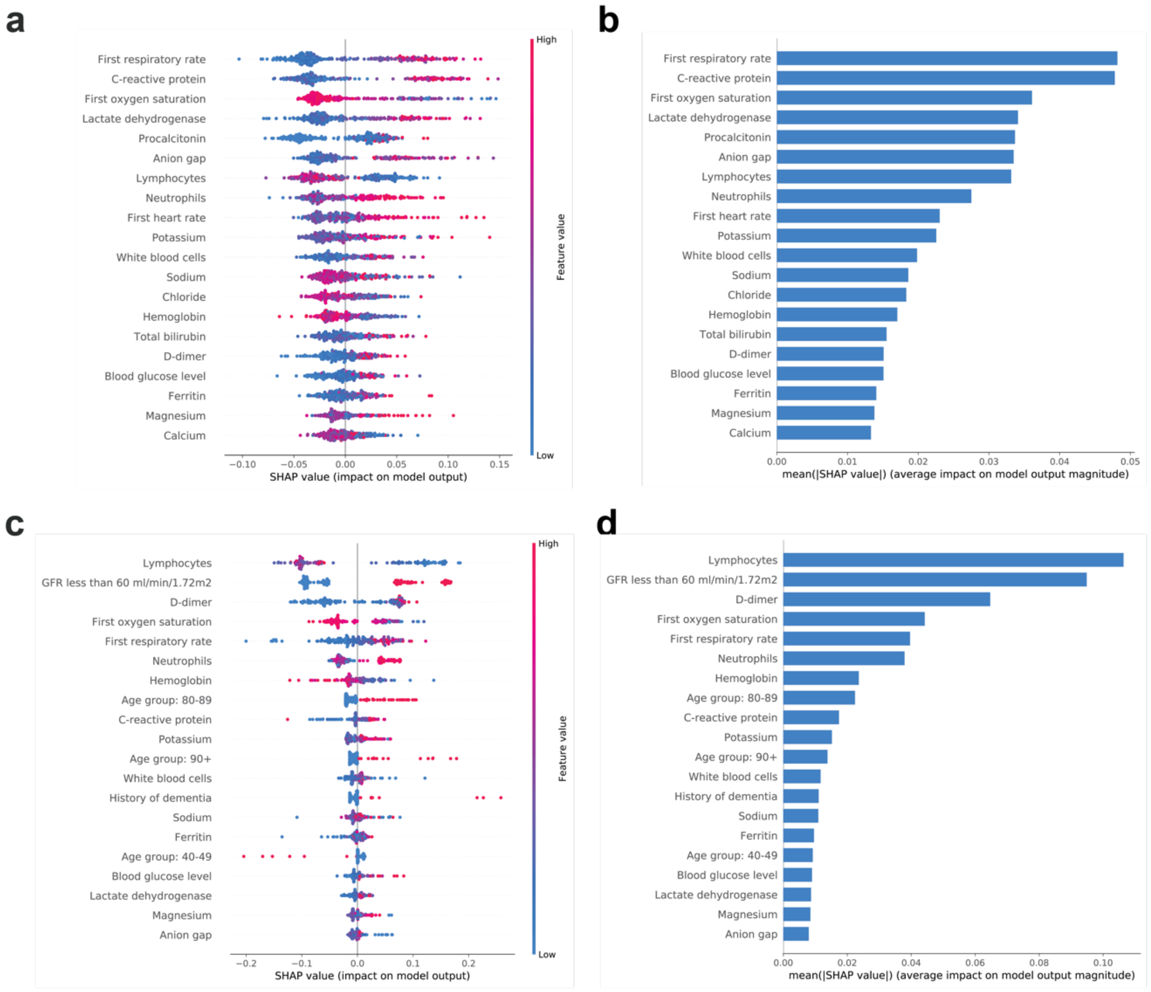

Supplementary figure 3. Variables of importance for ICU admission and mortality prediction in temporally distinct patients who encountered ED between May-August 2020. (A) SHAP value summary dot plot and (B) variable of importance of *RandomForest* algorithm-based ICU admission model. (C) SHAP value summary dot plot and (D) variable of importance of *RandomForest* algorithm-based mortality model.

Supplementary table 1. Selection of patients and variable details used for developing and testing the models

	Dataset used for model development<br>**March and April 2020**	Dataset used for external validation<br>**May to August 2020**
**Total number of patients with COVID-19 in multi-hospital Mass General Brigham database**	10826	8013
**Number of patients which visited emergency department**	3713	1754
**Number of patients remaining after removing missingness in outcome variable (ICU admission and mortality) and patients with incorrect entry in respiratory rate; knn imputation performed on these datasets**	3597	1711
**Number of patients included in the balanced dataset which was used for model development**	972 for ICU admission model and 688 for death prediction model	All 1711 patients were tested - unbalanced dataset

Variable category	**Variable used for model development and testing**
**Demographic**	age, sex, race, BMI
**Medication use**	ACEi/ARB, MRA, Calcium channel blocker, Betablocker, vasodilator, alphablocker, diuretic, antiplatelet, NSAID, Proton pump inhibitor, statin, anticoagulant
**History of past illness**	acute myocardial infarction, congestive heart failure, peripheral vascular disease, cerebrovascular disease, dementia, chronic obstructive pulmonary disease, rheumatic disease, peptic ulcer disease, mild liver disease, diabetes with or without complications, hemiplegia, renal disease, cancer, moderate/severe liver disease, metastatic cancer, AIDS, hypertension
**Clinical examination**	First respiratory rate, first heart rate, first oxygen saturation
**Laboratory values**	C-reactive protein, lymphocyte count, WBC count, neutrophil count, D-dimer, total bilirubin, lactate dehydrogenase, GFR, anion gap, hemoglobin, procalcitonin, glucose, alanine aminotransferase, serum sodium, calcium, magnesium, potassium, chloride and ferritin

Supplementary table 2. Risk factors identified for mortality and ICU admission in COVID-19 studies

Study authors	Study design	Period	Sample size	Risk factors for Mortality	Risk factor for ICU admission
**Elizabeth J Williamson et al.(*16*)**	Prospective cohort study	February - May 2020	17,278,392	Male - (HR) 1.59 (1.53-1.65),<br>Black - (HR) 1.48 (1.29-1.69),<br>BMI > 40- (HR) 1.92(1.72-2.13),<br>eGFR< 60- (HR) 1.33(1.28-1.40),<br>Diabetes- (HR) 1.90(1.72-1.96),<br>Chronic heart disease- (HR) 1.17(1.12-1.22),<br>Cancer < 1 year ago- (HR) 1.72 (1.50-1.96)	
**Christopher M Petrilli et al.(*34*)**	Prospective cohort study	March-April 2020	5279	NA	Heart failure- (OR) 1.9 (1.4-2.5),<br>BMI> 40- (OR) 1.5 (1.0-2.2),<br>Male Sex- (OR) 1.5 (1.3-1.8),<br>Oxygen saturation < 88% -(OR) 3.7 (2.8-4.8),<br>Troponin level > 1 (OR) 4.8(2.1-10.9),<br>CRP > 200- (OR) 5.1(2.8-9.2),<br>D-dimer > 2500-<br>(OR) 3.9 (2.6-6.0)
**Michaela R Anderson et al.(*35*)**	Retrospective study	March – April 2020	2466	BMI > 40 -(HR) 1.6 (1.1-2.1)	NA
**Sara Y Tartof et al.(*36*)**	Retrospective study	February-May 2020	6916	BMI 40-45 -(HR) 2.68 (1.43- 5.04),<br>BMI > 45- (HR) 4.18(2.12-8.26)	NA
**Matthew J Cummings et al.(*37*)**	Prospective cohort study	March-April 2020	1150	Older age- (HR) 1.31 (1.09-1.57) per 10 years increase,<br>Chronic cardiac disease – (HR) 1.76 (1.08-2.86),<br>Chronic pulmonary disease – (HR) 2.94 (1.48-5.84),<br>Higher concentrations of interleukin-6- (HR) 1.11 (1.02-1.20) per decile increase,<br>Higher concentrations of D-dimer- (HR) 1.11 (1.01-1.19) per decile increase	
**Shruti Gupta et al.(*38*)**	Retrospective cohort study	March - April 2020	2215	Older age (≥ 80 vs < 40 years- (OR) 11.15 (6.19-20.06),<br>Coronary artery disease- (OR) 1.47 (1.07-2.02),<br>Male- (OR) 1.50 (1.19-1.90),<br>BMI(≥ 40 vs < 25)- (OR) 1.51 (1.01-2.25),<br>Active cancer -(OR) 2.15 (1.35-3.43),<br>Pao2:Fio2 < vs ≥ 300 mm of Hg- (OR) 2.94 (2.11-4.08)	NA
**Giacomo Grasselli et al.(*39*)**	Retrospective cohort study	February-April 2020	3988	Older age -(HR) 1.75 (1.60-1.92),<br>Male sex- (HR) 1.57 (1.31-1.88),<br>High Fio2- (HR) 1.14 (1.10-1.19),<br>High PEEP (HR) 1.04 (1.01-1.06)<br>Type 2 DM-(HR) 1.18 (1.01-1.39)	NA
**Jonathan W.Cunningham et al.(*40*)**	Retrospective study	April -June 2020	63103	Morbid obesity- (OR) 2.30 (1.77-2.98),<br>Hypertension- (OR) 2.36 (1.79-3.12),<br>Male sex- (OR) 1.53 (1.20-1.95)	NA

Supplementary table 3. Description of machine learning algorithms

Model Type	Model name	Description
**Ensemble**	AdaBoostClassifier	It is a decision tree-based classifier where instead of a full tree, each tree only has one split, like a stump. Sequential ensembling is performed by boosting technique where an error that the first stump makes does impact the second stump. By fitting consecutive trees at every step, the accuracy increases than the previous tree. The drawback, however, is the chance of overfitting.
	BaggingClassifier	The bagging classifier fits base classifiers (default is decision tree) on random subsets of the training dataset and then average the individual predictions based on voting to form a final prediction. Here all variables are considered for splitting a node.
	GradientBoostingClassifier	In this machine learning technique, multiple weak prediction models are combined to form more robust prediction model. The negative gradient of the loss function (linked with the whole ensemble) is maximally correlated with weak learners.
	RandomForestClassifier	It is a decision tree-based classifier, which runs multiple trees in parallel. It uses a bagging technique to reduce variance, in which numerous subsets of data from the training sample are chosen randomly with replacement to train decision trees. The average of all predictions from decision trees is considered, which also reduces over-fitting. Unlike bagging classifier, here only a subset of features is considered at random and the best split is used.
	XGBClassifier	Extreme gradient boosting (XGB) classifier is similar to gradient boosting classifier, except that it uses second-order gradients of the loss function and uses advanced regularization, which improved model generalization. It is computed in parallel and also trains fast.
	ExtraTreesClassifier	It is an algorithm similar to Random forest classifier, where a subset of features is used but instead of considering the most discriminative threshold, randomly drawn thresholds are used. The best randomly generated threshold is then picked as the splitting rule.
**Gaussian process**	GaussianProcessClassifier	It implements Gaussian processes for probabilistic classification. It uses a latent function which allows a convenient formulation of model which is then removed during prediction.
**Linear models**	LogisticRegression	It is a machine learning algorithm in which the dependent categorical variable is predicted as a function of independent variables. Here regularization technique was used to prevent over-fitting.
	PassiveAggressiveClassifier	It is a linear model in which if prediction is correct, the model estimates are kept same but if prediction is incorrect, alterations are made in the model. They do not have a learning rate but they do have a regularization hyperparameter.
	SGDClassifier	It is a linear classifier that uses stochastic gradient descent as a solver and allows the usage a numerous loss function.
	Perceptron	It is also a linear model with stochastic gradient descent but is uses a set of functions to learn from inputs to ascertain if they belong to one class or the other.
**Naïve Bayes**	GaussianNB	It is a supervised machine learning algorithm which is based upon bayes theorem and it assumes that every feature is independent from values of other features for a given class.
**Nearest Neighbor**	KNeighborsClassifier	It is a machine learning algorithm where a user defined constant is considered for calculating the number of neighbors that can be grouped into one class. Here the distance between samples are generally calculated in terms of standard Euclidean distance.
**Support vector machine**	LinearSVC	It is a supervised machine learning algorithm where training data is fit to a model in which best fit hyperplanes divide the data into outcome categories.
**Tree based**	DecisionTreeClassifier	It is a non-parametric supervised machine learning method where a model is made to predict target variable by learning simple decision rules from the features provided in the training data.
**Discriminant analysis**	LinearDiscriminantAnalysis	It is a classifier algorithm with a linear decision boundary. The model is made by fitting class conditional densities to the training data. Gaussian distribution and similar covariance are the assumptions used by this algorithm.
	QuadraticDiscriminantAnalysis	It is a machine learning algorithm similar to Linear Discriminant analysis but with a quadratic decision boundary.
**Neural network**	MLPClassifier	MLPClassifier (multilayer perceptron classifier) is a type of neural network-based machine learning algorithm where the layers are formed using multiple layers (atleast three layers) of perceptron.

Supplementary table 4. Hyperparameters which were optimized for machine learning algorithms

Model Name	Hyperparameters optimized
**AdaBoostClassifier'**	'n_estimators': [10, 100, 1000, 10000],<br>'learning_rate': [0.001, 0.01, 0.1, 1]
**BaggingClassifier'**	'n_estimators': [10, 100, 1000, 10000],<br>'max_samples': [1, 10, 100, 1000]
**GradientBoostingClassifier'**	'n_estimators': [10, 100, 1000, 10000],<br>'learning_rate': [0.001, 0.01, 0.1, 1]
**RandomForestClassifier'**	'n_estimators': [10, 100, 1000, 10000],<br>'max_features': ['auto', 'log2'],<br>'max_depth': [2, 5, 10, 20, 50, 100],<br>'criterion': ['gini', 'entropy']
**XGBClassifier'**	'max_depth': [2, 5, 10, 20, 50, 100],<br>'n_estimators': [10, 100, 1000, 10000],<br>'learning_rate': [0.001, 0.01, 0.1, 1]
**ExtraTreesClassifier'**	'n_estimators': [100, 1000, 10000],<br>'criterion': ['gini', 'entropy'],<br>'max_features': ['auto', 'log2'],<br>'max_depth': [2, 5, 10, 20, 50, 100]
**GaussianProcessClassifier'**	'kernel': (RBF(length_scale=1), Matern(length_scale=1, nu=1.5), RationalQuadratic(alpha=1.5, length_scale=1))
**LogisticRegression'**	'penalty': ['l1', 'l2'],<br>'C': [0.1, 1, 10, 100, 200, 500, 1000]
**PassiveAggressiveClassifier'**	'C': [0.0001, 0.0003, 0.001, 0.003, 0.01],<br>'loss': ['hinge', 'squared_hinge'],<br>'n_iter_no_change': [5, 10, 30, 100, 300]
**SGDClassifier'**	'loss': ['modified_huber'],<br>'alpha': [0.01, 0.1, 0.5, 1],<br>'penalty': ['l2', 'l1', None]
**Perceptron'**	'alpha': [0.0001, 0.001, 0.01],<br>'penalty': ['l2', 'l1', None]
**GaussianNB'**	NA
**KNeighborsClassifier'**	'n_neighbors': [3, 5, 10, 20],<br>'leaf_size': [2, 5, 10, 20],<br>'p': [0.5, 1, 2, 5],<br>'weights': ['uniform', 'distance'],<br>'algorithm': ['auto', 'ball_tree', 'kd_tree', 'brute']
**LinearSVC'**	'penalty': ['l1', 'l2'],<br>'loss': ['hinge', 'squared_hinge'],<br>'C': [0.1, 0.5, 1, 5, 10]
**DecisionTreeClassifier'**	'criterion': ['gini', 'entropy'],<br>'splitter': ['random', 'best'],<br>'max_depth': [1, 2, 10],<br>'min_samples_leaf': [1, 2, 10]
**LinearDiscriminantAnalysis'**	'solver': ['svd', 'lsqr', 'eigen'],<br>'tol': [1e-05, 0.0001, 0.0003]
**QuadraticDiscriminantAnalysis'**	'reg_param': [0.1, 0.5, 0.7, 0.9],<br>'tol': [1e-05, 0.0001, 0.0003]
**MLPClassifier'**	'solver': ['lbfgs', 'adam'],

```
'learning_rate': ['constant', 'invscaling', 'adaptive'],
'hidden_layer_sizes': [(10, 7, 3), (30, 20, 12), (50, 35, 25), (70, 50, 35)],
'activation': ['identity', 'logistic', 'tanh', 'relu']
```

Supplementary table 5. Best hyperparameter values for machine learning algorithms that were chosen after tuning hyperparameters using *GridSearchCV* technique. F1 score used for tuning hyperparameters.

Method Type	Model name	Total fits tested	Best hyperparameter values for ICU admission prediction	Best hyperparameter values for death prediction
**Ensemble**	AdaBoostClassifier	80	'learning_rate': 0.01, 'n_estimators': 1000	'learning_rate': 0.001, 'n_estimators': 10000
	BaggingClassifier	80	'max_samples': 10, 'n_estimators': 10000	'max_samples': 100, 'n_estimators': 10000
	GradientBoostingClassifier	80	'learning_rate': 0.1, 'n_estimators': 100	'learning_rate': 0.1, 'n_estimators': 1000
	RandomForestClassifier	480	'criterion': 'entropy', 'max_depth': 10, 'max_features': 'log2', 'n_estimators': 100	'criterion': 'gini', 'max_depth': 2, 'max_features': 'log2', 'n_estimators': 100
	XGBClassifier	480	'learning_rate': 0.01, 'max_depth': 2, 'n_estimators': 1000	'learning_rate': 0.1, 'max_depth': 2, 'n_estimators': 100
	ExtraTreesClassifier	360	'criterion': 'entropy', 'max_depth': 20, 'max_features': 'auto', 'n_estimators': 1000	'criterion': 'entropy', 'max_depth': 5, 'max_features': 'log2', 'n_estimators': 100
**Gaussian process**	GaussianProcessClassifier	15	'kernel': RationalQuadratic(alpha=1.5, length_scale=1)	'kernel': RationalQuadratic(alpha=1.5, length_scale=1)
**Linear models**	LogisticRegression	70	'C': 200, 'penalty': 'l2'	'C': 100, 'penalty': 'l2'
	PassiveAggressiveClassifier	250	'C': 0.0001, 'loss': 'squared_hinge', 'n_iter_no_change': 10	'C': 0.0001, 'loss': 'hinge', 'n_iter_no_change': 100
	SGDClassifier	60	'alpha': 0.1, 'loss': 'modified_huber', 'penalty': 'l1'	'alpha': 0.01, 'loss': 'modified_huber', 'penalty': 'l1'
	Perceptron	45	'alpha': 0.01, 'penalty': 'l1'	'alpha': 0.01, 'penalty': 'l1'
**Naïve Bayes**	GaussianNB	5		
**Nearest Neighbor**	KNeighborsClassifier	2560	'algorithm': 'auto', 'leaf_size': 2, 'n_neighbors': 20, 'p': 1, 'weights': 'distance'	'algorithm': 'auto', 'leaf_size': 2, 'n_neighbors': 20, 'p': 1, 'weights': 'distance'
**Support vector machine**	LinearSVC	100	'C': 1, 'loss': 'squared_hinge', 'penalty': 'l2'	'C': 0.5, 'loss': 'squared_hinge', 'penalty': 'l2'
**Tree based**	DecisionTreeClassifier	180	'criterion': 'entropy', 'max_depth': 10, 'min_samples_leaf': 10, 'splitter': 'random'	'criterion': 'gini', 'max_depth': 10, 'min_samples_leaf': 10, 'splitter': 'random'
**Discriminant analysis**	LinearDiscriminantAnalysis	45	'solver': 'svd', 'tol': 1e-05	'solver': 'svd', 'tol': 1e-05
	QuadraticDiscriminantAnalysis	60	'reg_param': 0.9, 'tol': 1e-05	'reg_param': 0.1, 'tol': 1e-05
**Neural network**	MLPClassifier	480	'activation': 'logistic', 'hidden_layer_sizes': (30, 20, 12), 'learning_rate': 'constant', 'solver': 'adam'	'activation': 'identity', 'hidden_layer_sizes': (10, 7, 3), 'learning_rate': 'constant', 'solver': 'adam'

Supplementary table 6. Characteristics of patients who visited the emergency room between May and August 2020 for COVID-19, that were used to evaluate the machine learning models as a temporally distinct dataset. Variables stratified based on ICU admission and death of patients.

		**ICU admission**			**Death**		
	Overall	No	Yes	p	No	Yes	p
**n**	1711	1565	146		1633	78	
**Demographics**							
Age group (%)				<0.001			<0.001
0-9	48 (2.8)	46 (2.9)	2 (1.4)		48 (2.9)	0 (0.0)	
10-19	47 (2.7)	43 (2.7)	4 (2.7)		47 (2.9)	0 (0.0)	
20-29	208 (12.2)	201 (12.8)	7 (4.8)		207 (12.7)	1 (1.3)	
30-39	266 (15.5)	256 (16.4)	10 (6.8)		265 (16.2)	1 (1.3)	
40-49	216 (12.6)	208 (13.3)	8 (5.5)		215 (13.2)	1 (1.3)	
50-59	263 (15.4)	237 (15.1)	26 (17.8)		257 (15.7)	6 (7.7)	
60-69	277 (16.2)	241 (15.4)	36 (24.7)		266 (16.3)	11 (14.1)	
70-79	179 (10.5)	151 (9.6)	28 (19.2)		165 (10.1)	14 (17.9)	
80-89	151 (8.8)	135 (8.6)	16 (11.0)		122 (7.5)	29 (37.2)	
90+	56 (3.3)	47 (3.0)	9 (6.2)		41 (2.5)	15 (19.2)	
Sex = Male (%)	898 (52.5)	812 (51.9)	86 (58.9)	0.124	853 (52.2)	45 (57.7)	0.408
Race = Other (%)	1436 (83.9)	1327 (84.8)	109 (74.7)	0.002	1366 (83.6)	70 (89.7)	0.203
BMI_categorical (%)				0.087			<0.001
[0,25]	397 (23.2)	357 (22.8)	40 (27.4)		363 (22.2)	34 (43.6)	
(25,30]	672 (39.3)	627 (40.1)	45 (30.8)		646 (39.6)	26 (33.3)	
(30,75]	642 (37.5)	581 (37.1)	61 (41.8)		624 (38.2)	18 (23.1)	
**Medication use**							
On ACEi/ARB = TRUE (%)	302 (17.7)	270 (17.3)	32 (21.9)	0.193	283 (17.3)	19 (24.4)	0.15
On MRA = TRUE (%)	27 (1.6)	22 (1.4)	5 (3.4)	0.127	25 (1.5)	2 (2.6)	0.802
On Calcium channel blocker = TRUE (%)	171 (10.0)	144 (9.2)	27 (18.5)	0.001	156 (9.6)	15 (19.2)	0.01
On Betablocker = TRUE (%)	297 (17.4)	264 (16.9)	33 (22.6)	0.102	271 (16.6)	26 (33.3)	<0.001
On Vasodilator = TRUE (%)	77 (4.5)	70 (4.5)	7 (4.8)	1	69 (4.2)	8 (10.3)	0.026
On Alphablocker = TRUE (%)	37 (2.2)	36 (2.3)	1 (0.7)	0.324	35 (2.1)	2 (2.6)	1
On Diuretic = TRUE (%)	234 (13.7)	206 (13.2)	28 (19.2)	0.058	214 (13.1)	20 (25.6)	0.003
On Antiplatelet = TRUE (%)	34 (2.0)	31 (2.0)	3 (2.1)	1	32 (2.0)	2 (2.6)	1
On NSAIDs = TRUE (%)	255 (14.9)	242 (15.5)	13 (8.9)	0.045	249 (15.2)	6 (7.7)	0.095
On Proton pump inhibitor = TRUE (%)	328 (19.2)	296 (18.9)	32 (21.9)	0.44	303 (18.6)	25 (32.1)	0.005
On Statin = TRUE (%)	416 (24.3)	365 (23.3)	51 (34.9)	0.002	375 (23.0)	41 (52.6)	<0.001
On Anticoagulant = TRUE (%)	158 (9.2)	139 (8.9)	19 (13.0)	0.134	143 (8.8)	15 (19.2)	0.003
**History of past illness**							
Acute myocardial infarction = 1 (%)	24 (1.4)	23 (1.5)	1 (0.7)	0.687	18 (1.1)	6 (7.7)	<0.001
Congestive heart failure = 1 (%)	105 (6.1)	90 (5.8)	15 (10.3)	0.046	88 (5.4)	17 (21.8)	<0.001
Peripheral vascular disease = 1 (%)	69 (4.0)	58 (3.7)	11 (7.5)	0.042	61 (3.7)	8 (10.3)	0.01
Cerebrovascular disease = 1 (%)	103 (6.0)	86 (5.5)	17 (11.6)	0.005	90 (5.5)	13 (16.7)	<0.001
Dementia = 1 (%)	64 (3.7)	55 (3.5)	9 (6.2)	0.166	54 (3.3)	10 (12.8)	<0.001
Chronic obstructive pulmonary disease = 1 (%)	198 (11.6)	179 (11.4)	19 (13.0)	0.664	184 (11.3)	14 (17.9)	0.105

Rheumatic disease = 1 (%)	23 (1.3)	22 (1.4)	1 (0.7)	0.728	23 (1.4)	0 (0.0)	0.581
Peptic ulcer disease = 1 (%)	18 (1.1)	15 (1.0)	3 (2.1)	0.414	17 (1.0)	1 (1.3)	1
Mild liver disease = 1 (%)	72 (4.2)	63 (4.0)	9 (6.2)	0.31	68 (4.2)	4 (5.1)	0.9
Diabetes = 1 (%)	231 (13.5)	201 (12.8)	30 (20.5)	0.013	219 (13.4)	12 (15.4)	0.742
Diabetes with complications = 1 (%)	64 (3.7)	56 (3.6)	8 (5.5)	0.352	56 (3.4)	8 (10.3)	0.005
Hemiplegia = 1 (%)	17 (1.0)	14 (0.9)	3 (2.1)	0.36	16 (1.0)	1 (1.3)	1
Renal disease = 1 (%)	125 (7.3)	105 (6.7)	20 (13.7)	0.003	110 (6.7)	15 (19.2)	<0.001
Cancer = 1 (%)	101 (5.9)	91 (5.8)	10 (6.8)	0.746	88 (5.4)	13 (16.7)	<0.001
Moderate/severe liver disease = 1 (%)	7 (0.4)	7 (0.4)	0 (0.0)	0.895	7 (0.4)	0 (0.0)	1
Metastatic cancer = 1 (%)	10 (0.6)	8 (0.5)	2 (1.4)	0.463	8 (0.5)	2 (2.6)	0.112
AIDS = 1 (%)	5 (0.3)	5 (0.3)	0 (0.0)	1	5 (0.3)	0 (0.0)	1
Hypertension = 1 (%)	621 (36.3)	569 (36.4)	52 (35.6)	0.93	596 (36.5)	25 (32.1)	0.498
**Laboratory values and clinical examination**							
CRP (mg/L) (mean (SD))	59.67 (55.62)	54.74 (47.35)	112.57 (95.99)	<0.001	57.35 (52.74)	108.22 (85.05)	<0.001
First respiratory rate (counts/min) (mean (SD))	20.63 (5.61)	20.10 (4.87)	26.29 (8.93)	<0.001	20.37 (5.21)	26.03 (9.58)	<0.001
First heart rate (beats/min) (mean (SD))	93.66 (19.79)	92.94 (18.91)	101.34 (26.41)	<0.001	93.62 (19.71)	94.46 (21.51)	0.713
Sodium (mmol/L) (mean (SD))	137.93 (4.21)	138.05 (3.78)	136.59 (7.27)	<0.001	137.87 (3.98)	139.23 (7.52)	0.005
Calcium (mg/dL) (mean (SD))	9.19 (0.53)	9.21 (0.50)	8.99 (0.80)	<0.001	9.20 (0.53)	9.09 (0.61)	0.099
Magnesium (mg/dL) (mean (SD))	1.99 (0.25)	1.98 (0.22)	2.04 (0.44)	0.009	1.98 (0.22)	2.13 (0.53)	<0.001
Potassium (mmol/L) (mean (SD))	4.08 (0.55)	4.06 (0.50)	4.28 (0.90)	<0.001	4.06 (0.53)	4.40 (0.84)	<0.001
Chloride (mmol/L) (mean (SD))	99.86 (4.53)	100.04 (4.13)	97.92 (7.33)	<0.001	99.82 (4.31)	100.58 (7.92)	0.151
Lymphocytes (percentage; ref = 22-44%) (mean (SD))	21.96 (11.14)	22.61 (10.84)	15.02 (11.97)	<0.001	22.34 (10.83)	13.96 (14.33)	<0.001
Neutrophils (percentage; ref = 40-70%) (mean (SD))	66.76 (12.82)	66.04 (12.35)	74.40 (15.15)	<0.001	66.34 (12.48)	75.56 (16.34)	<0.001
WBC (x1000/µL) (mean (SD))	8.01 (4.14)	7.70 (3.42)	11.36 (7.98)	<0.001	7.82 (3.60)	11.93 (9.47)	<0.001
D-dimer (ng/mL) (mean (SD))	1572.09 (4557.18)	1356.21 (2340.48)	3886.22 (13414.10)	<0.001	1337.64 (2181.37)	6480.49 (18297.14)	<0.001
Total bilirubin (mg/dL) (mean (SD))	0.58 (0.94)	0.56 (0.89)	0.79 (1.32)	0.004	0.56 (0.84)	0.85 (2.08)	0.009
Ferritin (µg/L) (mean (SD))	545.29 (648.91)	508.27 (552.56)	942.05 (1224.49)	<0.001	534.84 (625.92)	763.94 (998.55)	0.002
LDH (Units) (mean (SD))	297.59 (142.72)	285.92 (118.19)	422.69 (268.92)	<0.001	293.93 (138.52)	374.34 (198.51)	<0.001
Low GFR (<60 ml/min/1.73m2) = TRUE (%)	361 (21.1)	288 (18.4)	73 (50.0)	<0.001	310 (19.0)	51 (65.4)	<0.001
Anion gap (mmol/L) (mean (SD))	14.57 (3.26)	14.32 (2.81)	17.29 (5.67)	<0.001	14.48 (3.11)	16.51 (5.18)	<0.001
Hemoglobin (g/dL) (mean (SD))	12.99 (1.89)	13.03 (1.83)	12.45 (2.36)	<0.001	13.03 (1.87)	12.11 (2.02)	<0.001
First O2 saturation (%) (mean (SD))	96.53 (4.13)	96.87 (3.43)	92.83 (7.67)	<0.001	96.74 (3.78)	92.04 (7.42)	<0.001
ventilator_use = TRUE (%)	NA	NA	NA	NA	58 (3.6)	21 (26.9)	<0.001
Procalcitonin (ng/ml) (mean (SD))	0.71 (3.80)	0.57 (2.71)	2.22 (9.39)	<0.001	0.63 (2.84)	2.56 (12.07)	<0.001
Glucose (mg/dL) (mean (SD))	139.28 (72.84)	134.96 (62.95)	185.62 (132.19)	<0.001	137.43 (68.93)	178.04 (124.64)	<0.001
ALT (IU/L) (mean (SD))	40.39 (50.55)	39.66 (49.28)	48.14 (62.31)	0.053	40.23 (48.83)	43.75 (78.83)	0.547

Supplementary table 7. Multiple comparison between ensemble methods and other types of machine learning algorithms using Two-stage linear step-up procedure of Benjamini, Krieger and Yekutieli test.

ICU admission models				
Multiple comparison of F1 scores				
**Two-stage linear step-up procedure of Benjamini, Krieger and Yekutieli**	**Mean rank diff.**	**Discovery?**	**q value**	**Individual P Value**
**Ensemble vs. Logistic regression**	22.17	Yes	0.0463	0.079
**Ensemble vs. Gaussian process**	37.17	Yes	0.0027	0.0032
**Ensemble vs. Other linear models**	50.13	Yes	<0.0001	<0.0001
**Ensemble vs. Naïve bayes**	42.87	Yes	0.0007	0.0007
**Ensemble vs. Nearest neighbor**	47.77	Yes	0.0002	0.0002
**Ensemble vs. Support vector machines**	55.37	Yes	<0.0001	<0.0001
**Ensemble vs. Tree-based**	17.97	No	0.0721	0.1545
**Ensemble vs. Discriminant analysis**	16.27	Yes	0.0463	0.0881
**Ensemble vs. Neural network**	23.37	Yes	0.0448	0.0641
**Mortality models**				
**Multiple comparison of PR AUC scores**				
**Two-stage linear step-up procedure of Benjamini, Krieger and Yekutieli**	**Mean rank diff.**	**Discovery?**	**q value**	**Individual P Value**
**Ensemble vs. Logistic regression**	33.18	Yes	0.0045	0.0085
**Ensemble vs. Gaussian process**	44.58	Yes	0.0003	0.0004
**Ensemble vs. Other linear models**	55.45	Yes	<0.0001	<0.0001
**Ensemble vs. Naïve bayes**	14.38	No	0.1001	0.2543
**Ensemble vs. Nearest neighbor**	52.38	Yes	<0.0001	<0.0001
**Ensemble vs. Support vector machines**	48.58	Yes	0.0001	0.0001
**Ensemble vs. Tree-based**	16.58	No	0.0849	0.1888
**Ensemble vs. Discriminant analysis**	2.433	No	0.2795	0.7986
**Ensemble vs. Neural network**	38.18	Yes	0.0016	0.0025

Supplementary table 8. Comparison of excluded patients of the majority class during model development. ICU admission and mortality models were developed using balanced datasets by random undersampling.

	**Patients included in ICU admission model development after random undersampling**				**Patients included in mortality model development after random undersampling**			
**n**	Overall	No	Yes	p	Overall	No	Yes	p
**Demographics**	3111	2625	486		3253	2909	344	
Age group (%)				0.796				0.307
0-9	33 (1.1)	31 (1.2)	2 (0.4)		33 (1.0)	30 (1.0)	3 (0.9)	
10-19	56 (1.8)	47 (1.8)	9 (1.9)		56 (1.7)	46 (1.6)	10 (2.9)	
20-29	322 (10.4)	274 (10.4)	48 (9.9)		337 (10.4)	296 (10.2)	41 (11.9)	
30-39	427 (13.7)	364 (13.9)	63 (13.0)		453 (13.9)	399 (13.7)	54 (15.7)	
40-49	520 (16.7)	426 (16.2)	94 (19.3)		562 (17.3)	501 (17.2)	61 (17.7)	
50-59	569 (18.3)	483 (18.4)	86 (17.7)		627 (19.3)	557 (19.1)	70 (20.3)	
60-69	420 (13.5)	353 (13.4)	67 (13.8)		514 (15.8)	464 (16.0)	50 (14.5)	
70-79	339 (10.9)	289 (11.0)	50 (10.3)		350 (10.8)	325 (11.2)	25 (7.3)	
80-89	275 (8.8)	231 (8.8)	44 (9.1)		230 (7.1)	208 (7.2)	22 (6.4)	
90+	150 (4.8)	127 (4.8)	23 (4.7)		91 (2.8)	83 (2.9)	8 (2.3)	
Sex = Male (%)	1537 (49.4)	1306 (49.8)	231 (47.5)	0.395	1650 (50.7)	1481 (50.9)	169 (49.1)	0.57
Race = Other (%)	2571 (82.6)	2164 (82.4)	407 (83.7)	0.526	2666 (82.0)	2374 (81.6)	292 (84.9)	0.156
BMI_categorical (%)				0.606				0.075
[0,25]	585 (18.8)	500 (19.0)	85 (17.5)		560 (17.2)	514 (17.7)	46 (13.4)	
(25,30]	1275 (41.0)	1078 (41.1)	197 (40.5)		1335 (41.0)	1196 (41.1)	139 (40.4)	
(30,75]	1251 (40.2)	1047 (39.9)	204 (42.0)		1358 (41.7)	1199 (41.2)	159 (46.2)	
**Medication use**								
On ACEi/ARB = TRUE (%)	544 (17.5)	463 (17.6)	81 (16.7)	0.651	584 (18.0)	516 (17.7)	68 (19.8)	0.394
On MRA = TRUE (%)	46 (1.5)	40 (1.5)	6 (1.2)	0.779	48 (1.5)	40 (1.4)	8 (2.3)	0.252
On Calcium channel blocker = TRUE (%)	368 (11.8)	308 (11.7)	60 (12.3)	0.758	369 (11.3)	338 (11.6)	31 (9.0)	0.176
On Betablocker = TRUE (%)	481 (15.5)	413 (15.7)	68 (14.0)	0.364	444 (13.6)	396 (13.6)	48 (14.0)	0.928
On Vasodilator = TRUE (%)	118 (3.8)	99 (3.8)	19 (3.9)	0.986	98 (3.0)	88 (3.0)	10 (2.9)	1
On Alphablocker = TRUE (%)	46 (1.5)	35 (1.3)	11 (2.3)	0.175	46 (1.4)	43 (1.5)	3 (0.9)	0.51
On Diuretic = TRUE (%)	433 (13.9)	365 (13.9)	68 (14.0)	1	404 (12.4)	365 (12.5)	39 (11.3)	0.577
On Antiplatelet = TRUE (%)	55 (1.8)	46 (1.8)	9 (1.9)	1	51 (1.6)	48 (1.7)	3 (0.9)	0.385
On NSAIDs = TRUE (%)	556 (17.9)	463 (17.6)	93 (19.1)	0.467	585 (18.0)	516 (17.7)	69 (20.1)	0.324
On Proton pump inhibitor = TRUE (%)	562 (18.1)	480 (18.3)	82 (16.9)	0.497	560 (17.2)	503 (17.3)	57 (16.6)	0.795
On Statin = TRUE (%)	801 (25.7)	674 (25.7)	127 (26.1)	0.877	783 (24.1)	713 (24.5)	70 (20.3)	0.101
On Anticoagulant = TRUE (%)	197 (6.3)	160 (6.1)	37 (7.6)	0.246	173 (5.3)	155 (5.3)	18 (5.2)	1
**History of past illness**								
Acute myocardial infarction = 1 (%)	54 (1.7)	41 (1.6)	13 (2.7)	0.124	43 (1.3)	41 (1.4)	2 (0.6)	0.307
Congestive heart failure = 1 (%)	195 (6.3)	159 (6.1)	36 (7.4)	0.305	153 (4.7)	136 (4.7)	17 (4.9)	0.931
Peripheral vascular disease = 1 (%)	109 (3.5)	89 (3.4)	20 (4.1)	0.507	92 (2.8)	81 (2.8)	11 (3.2)	0.791
Cerebrovascular disease = 1 (%)	170 (5.5)	148 (5.6)	22 (4.5)	0.378	140 (4.3)	129 (4.4)	11 (3.2)	0.353
Dementia = 1 (%)	124 (4.0)	102 (3.9)	22 (4.5)	0.591	90 (2.8)	83 (2.9)	7 (2.0)	0.483
Chronic obstructive pulmonary disease = 1 (%)	396 (12.7)	329 (12.5)	67 (13.8)	0.492	376 (11.6)	338 (11.6)	38 (11.0)	0.822
Rheumatic disease = 1 (%)	63 (2.0)	56 (2.1)	7 (1.4)	0.412	62 (1.9)	57 (2.0)	5 (1.5)	0.66
Peptic ulcer disease = 1 (%)	45 (1.4)	41 (1.6)	4 (0.8)	0.295	36 (1.1)	33 (1.1)	3 (0.9)	0.867
Mild liver disease = 1 (%)	145 (4.7)	124 (4.7)	21 (4.3)	0.787	150 (4.6)	139 (4.8)	11 (3.2)	0.236

Diabetes = 1 (%)	415 (13.3)	353 (13.4)	62 (12.8)	0.735	429 (13.2)	395 (13.6)	34 (9.9)	0.067
Diabetes with complications = 1 (%)	137 (4.4)	118 (4.5)	19 (3.9)	0.647	129 (4.0)	116 (4.0)	13 (3.8)	0.967
Hemiplegia = 1 (%)	12 (0.4)	11 (0.4)	1 (0.2)	0.765	14 (0.4)	11 (0.4)	3 (0.9)	0.375
Renal disease = 1 (%)	263 (8.5)	223 (8.5)	40 (8.2)	0.917	215 (6.6)	195 (6.7)	20 (5.8)	0.608
Cancer = 1 (%)	249 (8.0)	203 (7.7)	46 (9.5)	0.23	222 (6.8)	202 (6.9)	20 (5.8)	0.501
Moderate/severe liver disease = 1 (%)	9 (0.3)	6 (0.2)	3 (0.6)	0.314	10 (0.3)	9 (0.3)	1 (0.3)	1
Metastatic cancer = 1 (%)	23 (0.7)	18 (0.7)	5 (1.0)	0.601	16 (0.5)	15 (0.5)	1 (0.3)	0.876
AIDS = 1 (%)	17 (0.5)	15 (0.6)	2 (0.4)	0.917	15 (0.5)	13 (0.4)	2 (0.6)	1
Hypertension = 1 (%)	1113 (35.8)	944 (36.0)	169 (34.8)	0.652	1169 (35.9)	1053 (36.2)	116 (33.7)	0.398
**Laboratory values and clinical examination**								
CRP (mg/L) (mean (SD))	63.74 (50.23)	64.20 (50.53)	61.24 (48.55)	0.232	69.74 (58.33)	69.97 (58.25)	67.75 (59.05)	0.505
First respiratory rate (counts/min) (mean (SD))	20.90 (5.10)	20.97 (5.14)	20.56 (4.89)	0.108	21.25 (5.61)	21.26 (5.66)	21.15 (5.12)	0.725
First heart rate (beats/min) (mean (SD))	93.64 (17.77)	93.75 (17.81)	93.04 (17.53)	0.416	94.79 (18.00)	94.78 (18.07)	94.86 (17.44)	0.94
Sodium (mmol/L) (mean (SD))	137.97 (3.87)	137.94 (3.90)	138.13 (3.72)	0.303	137.63 (3.81)	137.63 (3.89)	137.65 (3.05)	0.93
Calcium (mg/dL) (mean (SD))	9.06 (0.47)	9.06 (0.47)	9.07 (0.45)	0.746	9.04 (0.47)	9.04 (0.47)	9.04 (0.41)	0.849
Magnesium (mg/dL) (mean (SD))	2.01 (0.22)	2.01 (0.22)	2.00 (0.22)	0.452	2.01 (0.23)	2.01 (0.23)	2.01 (0.22)	0.784
Potassium (mmol/L) (mean (SD))	4.02 (0.46)	4.03 (0.46)	4.00 (0.46)	0.246	4.01 (0.46)	4.01 (0.45)	3.99 (0.49)	0.508
Chloride (mmol/L) (mean (SD))	99.62 (4.21)	99.59 (4.25)	99.79 (3.98)	0.341	99.22 (4.27)	99.21 (4.35)	99.34 (3.57)	0.576
Lymphocytes (percentage; ref = 22-44%) (mean (SD))	20.66 (9.26)	20.59 (9.29)	21.06 (9.09)	0.31	20.31 (9.16)	20.28 (9.15)	20.59 (9.27)	0.547
Neutrophils (percentage; ref = 40-70%) (mean (SD))	68.33 (11.06)	68.38 (11.08)	68.06 (10.98)	0.547	68.91 (11.02)	68.94 (11.02)	68.70 (11.04)	0.712
WBC (x1000/μL) (mean (SD))	6.87 (3.65)	6.90 (3.82)	6.72 (2.55)	0.306	6.96 (3.69)	6.98 (3.83)	6.75 (2.19)	0.258
D-dimer (ng/mL) (mean (SD))	1364.85 (1818.32)	1384.53 (1880.51)	1258.51 (1433.53)	0.16	1388.79 (2328.01)	1401.10 (2372.08)	1284.72 (1914.97)	0.381
Total bilirubin (mg/dL) (mean (SD))	0.50 (0.60)	0.51 (0.64)	0.47 (0.25)	0.189	0.51 (0.59)	0.51 (0.62)	0.48 (0.26)	0.304
Ferritin (μg/L) (mean (SD))	592.91 (674.79)	601.21 (706.13)	548.08 (468.69)	0.111	631.31 (746.44)	632.36 (736.93)	622.37 (823.61)	0.814
LDH (Units) (mean (SD))	304.65 (164.23)	304.59 (169.31)	304.99 (133.71)	0.96	314.29 (168.73)	314.98 (174.37)	308.44 (110.09)	0.496
Low GFR (<60 ml/min/1.73m2) = TRUE (%)	642 (20.6)	550 (21.0)	92 (18.9)	0.342	593 (18.2)	539 (18.5)	54 (15.7)	0.225
Anion gap (mmol/L) (mean (SD))	14.81 (2.55)	14.84 (2.59)	14.61 (2.33)	0.06	14.92 (2.66)	14.93 (2.72)	14.82 (2.17)	0.472
Hemoglobin (g/dL) (mean (SD))	13.25 (1.71)	13.23 (1.72)	13.34 (1.66)	0.23	13.33 (1.65)	13.34 (1.67)	13.28 (1.51)	0.543
First O2 saturation (%) (mean (SD))	96.43 (3.78)	96.42 (3.86)	96.49 (3.29)	0.712	96.09 (4.55)	96.08 (4.65)	96.22 (3.61)	0.594
ventilator_use = TRUE (%)					260 (8.0)	231 (7.9)	29 (8.4)	0.833
Procalcitonin (ng/ml) (mean (SD))	0.62 (3.21)	0.65 (3.44)	0.43 (1.34)	0.153	0.64 (3.28)	0.61 (2.96)	0.84 (5.29)	0.22
Glucose (mg/dL) (mean (SD))	134.41 (56.23)	134.92 (56.66)	131.68 (53.81)	0.242	137.23 (63.00)	137.73 (64.71)	132.98 (45.93)	0.187
ALT (IU/L) (mean (SD))	38.63 (31.42)	38.93 (32.51)	36.98 (24.68)	0.208	40.22 (32.76)	39.87 (32.77)	43.18 (32.56)	0.076
