# Supplementary material for: Comparing machine learning algorithms for predicting ICU admission and mortality in COVID-19
Source: NPJ Digit Med. 2021 May 21;4:87. doi: 10.1038/s41746-021-00456-x (PMC8140139; doi:10.1038/s41746-021-00456-x)
Supplement: Supplementary file 2 — Reporting Summary [file 41746_2021_456_MOESM2_ESM.pdf]

nature research

Corresponding author(s): Sayon Dutta, MD and Rakesh K. Jain, PhD

Last updated by author(s): Apr 15, 2021

Reporting Summary

Nature Research wishes to improve the reproducibility of the work that we publish. This form provides structure for consistency and transparency in reporting. For further information on Nature Research policies, see our Editorial Policies and the Editorial Policy Checklist.

Statistics

For all statistical analyses, confirm that the following items are present in the figure legend, table legend, main text, or Methods section.

n/a	Confirmed	
☐	☒	The exact sample size (*n*) for each experimental group/condition, given as a discrete number and unit of measurement
☐	☒	A statement on whether measurements were taken from distinct samples or whether the same sample was measured repeatedly
☐	☒	The statistical test(s) used AND whether they are one- or two-sided<br>*Only common tests should be described solely by name; describe more complex techniques in the Methods section.*
☐	☒	A description of all covariates tested
☐	☒	A description of any assumptions or corrections, such as tests of normality and adjustment for multiple comparisons
☐	☒	A full description of the statistical parameters including central tendency (e.g. means) or other basic estimates (e.g. regression coefficient) AND variation (e.g. standard deviation) or associated estimates of uncertainty (e.g. confidence intervals)
☐	☒	For null hypothesis testing, the test statistic (e.g. *F*, *t*, *r*) with confidence intervals, effect sizes, degrees of freedom and *P* value noted<br>*Give P values as exact values whenever suitable.*
☒	☐	For Bayesian analysis, information on the choice of priors and Markov chain Monte Carlo settings
☐	☒	For hierarchical and complex designs, identification of the appropriate level for tests and full reporting of outcomes
☒	☐	Estimates of effect sizes (e.g. Cohen's *d*, Pearson's *r*), indicating how they were calculated

Our web collection on statistics for biologists contains articles on many of the points above.

Software and code

Policy information about availability of computer code

Data collection	Data cleaning and processing were performed with R (R Core Team, version 3.6.3) using the tidyverse and comorbidity packages (for cross referencing ICD-10 codes in each comorbidity to Charlson method).
Data analysis	Machine learning model development was done using Python (details in Supplementary Methods). The programming code for R and Python are available upon request addressed to the corresponding authors: Sayon Dutta, MD (sdutta1@partners.org) and Rakesh K. Jain, PhD (jain@steele.mgh.harvard.edu).

For manuscripts utilizing custom algorithms or software that are central to the research but not yet described in published literature, software must be made available to editors and reviewers. We strongly encourage code deposition in a community repository (e.g. GitHub). See the Nature Research guidelines for submitting code & software for further information.

Data

Policy information about availability of data

All manuscripts must include a data availability statement. This statement should provide the following information, where applicable:

- Accession codes, unique identifiers, or web links for publicly available datasets
- A list of figures that have associated raw data
- A description of any restrictions on data availability

The clinical data used in this study belongs to MGB healthcare and restrictions apply to the availability of these data. Qualified researchers affiliated with the Mass General Brigham (MGB) may apply for access to these data through the MGB institutional review board.

Field-specific reporting

Please select the one below that is the best fit for your research. If you are not sure, read the appropriate sections before making your selection.

☒ Life sciences ☐ Behavioural & social sciences ☐ Ecological, evolutionary & environmental sciences

For a reference copy of the document with all sections, see nature.com/documents/nr-reporting-summary-flat.pdf

Life sciences study design

All studies must disclose on these points even when the disclosure is negative.

Sample size	This study was performed using patient data available in the MGB database. We selected all COVID-19 patients that presented to the Emergency department (n = 3,597 for training cohort and n = 1,711 for temporal-validation cohort) and developed machine learning models.
Data exclusions	We excluded patients which did not have information about outcome variable - which in our study was ICU admission or mortality.
Replication	We used stratified KFold method (during cross validation) for measuring the performance of machine learning models.
Randomization	For balancing the datasets, we performed random undersampling. To make sure that there was no bias during undersampling, we compared the excluded patients of the majority class with that of the included patients (Supplementary table 8). There were no covariate which differed significantly between these two groups suggesting that undersampling did not introduce any bias.
Blinding	Since this machine learning study was done by collecting past clinical data, we collected both independent and dependent variables from the database. Therefore blinding is not applicable to this study design.

Reporting for specific materials, systems and methods

We require information from authors about some types of materials, experimental systems and methods used in many studies. Here, indicate whether each material, system or method listed is relevant to your study. If you are not sure if a list item applies to your research, read the appropriate section before selecting a response.

Materials & experimental systems

n/a	Involved in the study	
☒	☐	Antibodies
☒	☐	Eukaryotic cell lines
☒	☐	Palaeontology and archaeology
☒	☐	Animals and other organisms
☒	☐	Human research participants
☐	☒	Clinical data
☒	☐	Dual use research of concern

Methods

n/a	Involved in the study	
☒	☐	ChIP-seq
☒	☐	Flow cytometry
☒	☐	MRI-based neuroimaging

Clinical data

Policy information about clinical studies

All manuscripts should comply with the ICMJE guidelines for publication of clinical research and a completed CONSORT checklist must be included with all submissions.

Clinical trial registration	N/A
Study protocol	N/A - since this is not a clinical trial.
Data collection	Data was collected from patients which presented to the ED with COVID-19 between March till August 2020.
Outcomes	ICU admission within 5 days and mortality within 28 days due to COVID-19
